# Biomaterials Used for Periodontal Disease Treatment: Focusing on Immunomodulatory Properties

**DOI:** 10.1155/2022/7693793

**Published:** 2022-04-26

**Authors:** H. Garzón, L. J. Suárez, S. Muñoz, J. Cardona, M. Fontalvo, C. A. Alfonso-Rodríguez

**Affiliations:** ^1^Grupo de Investigación en Salud Oral, Departamento de Periodoncia, Universidad Antonio Nariño, Bogotá, Colombia; ^2^Departamento de Ciencias Básicas y Medicina Oral, Universidad Nacional de Colombia, Bogotá, Colombia

## Abstract

The growing use of biomaterials with different therapeutic purposes increases the need for their physiological understanding as well as to seek its integration with the human body. Chronic inflammatory local pathologies, generally associated with infectious or autoimmunity processes, have been a current therapeutic target due to the difficulty in their treatment. The recent development of biomaterials with immunomodulatory capacity would then become one of the possible strategies for their management in local pathologies, by intervening in situ, without generating alterations in the systemic immune response. The treatment of periodontal disease as an inflammatory entity has involved the use of different approaches and biomaterials. There is no conclusive, high evidence about the use of these biomaterials in the regeneration of periodontitis sequelae, so the profession keeps looking for other different strategies. The use of biomaterials with immunomodulatory properties could be one, with a promising future. This review of the literature summarizes the scientific evidence about biomaterials used in the treatment of periodontal disease.

## 1. Introduction

The “immunomodulation processes” were initially framed as the possibility of manipulating the immune system, vaccines becoming one of the most classic examples [1]. This possibility of managing the immune system for anti-infective purposes initially opened the door to different strategies that would allow it to become a preventive and therapeutic focus [2]. Faced with this, biomaterials are then presented as one of the alternatives through which manipulation of the immune system is possible [3]. Technological advances have allowed the fabrication process of biomaterials to be controlled and so strictly accurate that the possibility of adverse reactions is minimum. In addition, the specificity that confers punctual therapeutic targets diminishes in turn the possibility of immunological adverse effects that affect the host function against antigens [4].

In the oral environment, there are a lot of biomaterials used constantly for restorative or anti-infectious purposes [5], being periodontal disease one of the main targets for a treat. Periodontal disease is a chronic inflammatory entity caused by the accumulation of biofilms on the tooth surface and their interaction with the immune system of the host [6]. The proposed treatment for this pathology has been mainly the classic anti-infectious management through scaling and root planning consisting of mechanical removal of the dental biofilm. In some cases, the administration of systemic antibiotics according to the severity could be advised [7].

In addition to the previous one, other approaches have been proposed, for example, the use of anti-inflammatories. Arachidonic acid is, among others, a mediator of various proinflammatory cytokines associated with bone loss [8], so to intervene with its actions with the systemic use of anti-inflammatory medications mainly NSAIDs (nonsteroidal anti-inflammatory drugs) have been proposed, but with inconclusive results in the literature [9]. On the other hand, the consequences at the gastrointestinal level, such as gastritis or colitis, are emphasized due to its prolonged use, and it does not represent significant periodontal clinical changes either, compared with the conventional treatment of scaling and root planning [10]. At the local level, they have also been placed in the gingival sulcus, but the vehicles and concentrations are ineffective, and the medication ends up lost without significant clinical changes [11].

Understanding the immunopathological process of periodontal disease, thinking about an immunomodulatory approach would have a place in its current treatment [12]. However, as the consumption of oral immunomodulatory drugs has limitations to achieve effective concentrations in the gingival sulcus, in addition to lack of specificity when administered systemically [13], the biomaterials designed to release therapeutic compounds directly into the sulcus would face the same problems in addition to the physical conditions of the oral environment that challenge the maintenance and controlled degradation of the biomaterial containing.

Given the impossibility of complete periodontal regeneration after treatment [14], it is necessary to refocus efforts toward the development of a biomaterial with the controlled release, of among others, immunomodulatory drugs. It would then become something useful for controlling the disease more effectively and treating the secondary effects of periodontitis and its treatment, maintaining the tooth for prolonged periods, and avoiding the use of artificial prosthetic abutments for dental replacement.

## 2. Immunomodulation with Biomaterials

The chronic inflammatory pathologies represent a global challenge implying an altered or perpetuated function of the immune system. Faced with the complexity of a treatment associated with the repercussions on the systemic immune response and in other organs, the development of biomaterials represents an opportunity to reunderstand and refocus efforts in search of a controlled treatment with no systemic repercussions [15]. The development of biomaterials needs the confluence of health sciences and engineering because not only it is not enough to understand the biological processes associated with the implantation of the biomaterial but also how it should be designed, in addition to being biocompatible, and to the control of the mechanical properties [16].

The endless possibilities for fabrication and functionalization of biomaterials confer a milliard of preventive and therapeutic uses [17]. But a therapeutic objective has been described as one of the pillars under which the design process of biomaterials must be focused [18]. To achieve this goal, it is necessary to consider different parameters, ranging from the design to its implementation: the shape taken by the biomaterial (solid, hydrogel, or micro/nanoparticles), the degree of molecular bonding, degradation, hydrophobicity or hydrophilicity, topography, and its nature (natural or synthetic derivative) [19].

Looking for immunomodulation of the host that interferes with the disease and allows its treatment with effective tissue regeneration, many different strategies have been developed [20]. These strategies include the following.

### 2.1. Surface Modifications

The placement of films and brushes of a dense hydrophilic polymer functions as surface modification of the biomaterial to protein adsorption [21]. In this way, cell behavior, leukocyte activation, and, therefore, foreign body reactions are modified [22]. Another example is the use of hydrogels that function as a coating of the material, associated with their properties such as high-water content, ease of transport of solutes, and having different active groups that can be chemically modified [22]. Surface biochemistry also influences the adhesion of macrophages and their secretory cytokine profile [23].

Neutral hydrophilic type polymers have been shown to promote reduced macrophage activation and reduced giant cell formation compared to ionic surfaces [24]. Isolating the biomaterial using different coatings to avoid an immunological reaction has shown satisfactory results in vitro. However, follow-up studies during chronic phases of inflammation have reported a lack of stability of the coating over time and the possibility of generating a foreign body reaction [25].

### 2.2. Use of Biomimetic Components of the Extracellular Matrix

The structure and composition of the material are very important in relating to cell viability and avoiding undesirable adverse reactions. It is necessary to recreate an extracellular matrix that will generate a microenvironment very similar to a normal one to promote adequate healing [26, 27]. Different strategies have been devised to obtain it, one of which is the removal of cells from the extracellular matrix (ECM) since it can be used to recreate a proregenerative environment [28]. An example of this is the conditioning with some glycosaminoglycans, such as sulfated hyaluronan capable of inducing a cellular response guided towards M2 macrophages producers of IL-10, which prevents an exacerbated inflammatory response [29].

This response of differentiation towards M2 macrophages is currently a focus of attention because macrophages dichotomy into M1 or M2 profiles plays a key role in the development of foreign body or severe inflammatory reactions. Ideally, the differentiation would be sought towards M2, described as a predictor in the reparative and integration field of the biomaterial [30].

Another chemical modification of the biomaterial is the removal of the cells or decellularization to obtain scaffolds. This involves the removal of certain components with immunogenic capacity: cellular genetic material, cell membrane antigens, and MHC. This leads to good tolerability to the biomaterial, in addition to being able to contain immunomodulatory cytokines and some growth factors. However, achieving the complete removal of all cells is a difficult task [31].

### 2.3. Hydrogels for the Isolation of Encapsulated Therapeutic Cells or Drugs

The use of semipermeable hydrogels prevents direct contact with the cells of the immune system; however, some small molecules, such as oxygen free radicals and cytokines, can cross this type of hydrogels [32]. In general, gels have been used constantly by having the advantage of remaining stable in the area [33]. They have also been constituted as a new form of drug release in a controlled manner, and additionally, the incorporation of TRAPS is to encapsulate proinflammatory signals such as TNF-*α* [34].

Controlled release of drugs becomes another strategy to be applied to immunomodulation. Different substances such as dexamethasone [35, 36], heparin [37], and superoxide dismutase [38] have been placed in different coatings and reservoirs of biomaterials, decreasing the inflammatory processes and the encapsulation of the material itself. However, by resorting to the pharmacokinetics of these drugs, the concentration and bioactivity are lost over time, associated with the time required for the degradation of the material. Other strategies, such as the use of nanoparticles coated with certain medications, have shown a slower and more controlled release of the drug [39].

The direct incorporation of cytokines such as TGF-*β* and IL-10 into hydrogels has been the current therapeutic focus. One of the limitations reported for this use is the maintenance of the cytokine in the time since being exposed to the environment, which makes it eventually degradable in undesired short periods [40]. Another strategy used in bioengineering for the controlled liberation of medications is the use of multilayer coatings with electrolytes; this makes ease the handling of thickness and the placement of different molecules possible [41].

### 2.4. Modifying the Physical Properties of the Interface with the Organism

The physical properties of the biomaterial, such as roughness, topography, and geometry, play a very important role in the adsorption and the cellular immune response. Topographic changes in the nanometric scale can modify the activity of protein adsorption. It has been reported that direct influence on cells can be achieved on surfaces with microcharacteristics of 10–100 *μ*m [42]. When the cells can be elongated, thanks to the topographic confirmation, in an animal model, a development of the M2 profile and the reduction of proinflammatory cytokines are reported. This is explained under the principle that the topographic modification favors this dimensional and morphological change of the macrophage which allows the development of an anti-inflammatory profile [43].

The engineering of surfaces then becomes a viable alternative to think about modifying cellular processes, under the following three main characteristics: changing the hydrophilicity of the surface, changing the nano and microtopography, and controlling the electric charge of the surface; those interventions would have a clear influence on the immune response to biomaterials [44]. The structure of the biomaterial has been one of the focal points of attention because, in addition to chemistry, the topography of the biomaterial must allow, among others, cell adhesion.

An example of this can be titanium dental implants. Titanium is characterized not only by its biocompatibility but also by its inherent immunogenic capacity. The exacerbated inflammatory process associated with the placement of dental implants has been considered one of their failure factors. In this way, different strategies have been sought to modify this structure. One of these would be the anodization of the titanium surface for the formation of nanotubes of titanium dioxide causing a lower release of proinflammatory factors. This also translates into less migration and recruitment of macrophages in the area. If this union does not exist, an immunological process would not be initiated [45].

### 2.5. Periodontal Disease Treatment and Biomaterials

Chronic periodontitis is an inflammatory disease of the supporting tissues of the tooth, in response to a microbial biofilm that leads to the activation of the immune system. Uncontrolled inflammation facilitated the progression of disease [46]. The whole process from the beginning to the outcome is determined and regulated by the host [47]. The immunoinflammatory response in periodontal disease should not be seen as a one-dimensional process that permits alveolar destruction, but rather as an interactive multidimensional process, constantly adjusted to local and systemic needs and conditions.

By the 1960s, the immune response was mentioned as the fundamental axis of the onset and progression of periodontal disease. The presence of anticollagen antibodies produced by plasma cells in the periodontal tissue of patients with periodontal disease was demonstrated [48]. It would then be one of the most important antecedents, where a direct relationship of the action of the immune system with the progression of periodontal disease is established.

The treatment of periodontal disease has different perspectives: the first one is the scaling and root planning, consisting of the mechanical removal of bacterial biofilm on the root surface. It turns out to be an operator-dependent treatment since it is necessary to remove the plaque from the deepest surface of the periodontal pocket to obtain the desired effect. Being a partially effective treatment, currently, the therapeutic approach to periodontal disease needs to be reunderstood and redefined in the spot of the disease's nature, as a process consistent with a loss of immunological regulation [49].

Periodontal disease management includes, in addition to the performance of scaling and root planning, therapies such as the systemic administration of antibiotics [7]; however, once the dental biofilm is mechanically removed, the inflammation ceases, and therefore, the disease can be controlled for a specific period. Even so, there is no way to know when the disease reactivates, so its control is subjected to the individual's response and individual oral hygiene [50, 51]. However, two inevitable “sequelae” have been described after periodontal treatment: the first is a process of periodontal repair with the formation of a long junctional epithelium [14]. The second would be gingival recessions that structurally and aesthetically affect subsequent oral rehabilitation processes.

Faced with the advent of dental biomaterials and tissue engineering, for more than one decade now, the approach to the management of this disease sequelae has brought new challenges in the search for the regeneration of the periodontal tissues [52]. The use of different biomaterials that serve as scaffolds for growth factors and medications to promote periodontal regeneration has been tried for some years. However, the conditions of the oral microenvironment make it difficult to achieve definitive results. The nature of periodontal tissues possesses an important challenge: first, because it has three different embryological origins, and second, conditions such as humidity, pH, and temperature make the scaffolding difficult to fix or achieve a programmed release and/or effective controlled medication [53].

Natural and synthetic origin biomaterials have been studied, but regeneration of periodontal defects persists to be a field with many difficulties, reporting just preliminary results and limited gain in terms of true regeneration of the support and protection apparatus [54]. Then, the use of therapies with controlled release of drugs loaded in different materials such as hydrogels, biolayers, micro, and nanofibers has been developed and has promising results [55–57].

### 2.6. Natural Hydrogels

Hydrogels can behave structurally as an extracellular matrix because of their micro/nanostructure. Natural polymers have been tested including collagen, alginate, chitosan, and fibrin, having different functions according to the place where placed. In periodontal disease, cell migration, proliferation, and differentiation are reported with their use. The most common material used in hydrogels is collagen, with different degrees of porosity, together with the loading with cells of the periodontal ligament; results have shown that with a porosity of 80% after 2 weeks of culture, cells like those of the periodontal ligament are generated [58]. Blended hydrogels composed of polyvinyl alcohol (PVA) and collagen also demonstrated their potential as membranes for guiding periodontal tissue regeneration [59].

Another example would be referred to as the use of chitosan hydrogels combined with beta-glycerol phosphate and nanohydroxyapatite, which have been used for alveolar regeneration, because they are attributed to osteogenic properties [36, 60].

Despite the satisfactory results, two major limitations for the use of hydrogels are reported: rapid degradation, which is a great obstacle to the regeneration of stable tissues, and second, the obtention process and cost that are still considerably complex and higher, compared to synthetic polymers [61].

### 2.7. Synthetic Hydrogels

Polymer-based hydrogels used for periodontal treatment can be summarized in the use of polyethylene glycol (PEG), polylactic acid (PLA), polyglycolic acid (PGL), and their copolymers. These have better primary stability, better mechanical properties, and a better three-dimensional conformation for cellular development, compared to natural hydrogels [62]. The plasticity offered by this type of polymer allows them to be combined in different proportions to improve some properties, whether chemical, physical, mechanical, or biological [38].

An example of this would be the combination between PLA and PGL described almost a decade ago, which makes it possible to control the degradation times of the material, and its hydrophilicity, in addition, to permitting the implantation of molecules that favor cell adhesion, functioning as biomimetic scaffolds [63]. The results are promising for bone regeneration [64], regeneration of periodontal ligament cells [65], and mineralization of cementoblasts [66]. However, complete regeneration of the dentoalveolar and dentogingival complex has not been possible.

### 2.8. Micro/Nanoparticles

Its use has been proposed for the release of tetracycline and statins [67]. These microparticles have been also used as a component of toothpaste or applied directly in the oral cavity, but it has some limitations to the controlled release of the drug. Although they are easy to manufacture, they have little control of drug release, low dispersion, and instability in aqueous media. Nanotechnology has presented solutions such as nanoencapsulation of the drug, which prevents premature degradation in its interaction with the biological environment, favoring absorption in a particular tissue, bioavailability, retention time, and can improve its entry into cells [68]. Not only drugs can be included in these “controlled release” biomaterials but also any kind of molecule can be included, opening the door for the use of immunomodulatory drugs. Some authors suggest that some mediators like IFN-*γ* as immunoregulatory molecules could be a therapeutic target in periodontal disease, but the evidence in this field is still inconclusive because of their dual action in an inflammatory microenvironment [69].

It has been reported that these nanoparticles are stable in blood, nontoxic, nonthrombogenic, noninflammatory, or immunogenic, biodegradable, do not activate neutrophils, can evade the reticuloendothelial system, and can apply to several molecules such as drugs, proteins, peptides, and nucleic acids [70]. They have been used for the release of different drugs such as minocycline in an animal model in dogs, with different concentrations [71]. The encapsulation of tetracycline nanoparticles in chitosan matrices is tested, reporting that the dispersion of these in this matrix can be more homogeneous, adapts anatomically, and can have a more controlled and homogeneous release of the drug [72].

### 2.9. Biofilms

They are matrices where it is possible to distribute a drug that can be applied directly or with the use of a solvent. These can be easily applied in the oral cavity, cheeks, or even on the gingival surface. The release of this drug occurs by diffusion (in the case of being insoluble in water and having a nondegradable polymer) or by the dissolution of the matrix or erosion (in the case of biodegradable polymeric materials). One of the advantages of this material is that it is minimally invasive [73]. In the oral microenvironment, the existence of biofilms is the most difficult problem to overcome in therapeutic plans because of their high resistance to antibiotics or other drugs. Some biomaterials have been developed to interrupt the biofilm formation or affect the mature biofilm, an example is a Cu/CaOH_2_-based endodontic paste against *S. aureus*, *P. aeruginosa*, and *C. Albicans*, with promising in vitro results [5].

Some polymeric materials such as PLA, PLGA, collagen, alginate, and chitosan have been used as matrices that contain factors for biofilm disruption. Various drugs have been incorporated for periodontal treatment such as tetracycline, metronidazole, meloxicam, and amoxicillin. An example of this is the currently marketed PerioChip. However, the results are not significantly different from the conventional treatment, and in the same way, the high costs of each chip would not compensate for the cost-benefit of its application in the oral cavity [74].

### 2.10. Micro/Nanofibers

Fibers are another type of biomaterial that has been used in the oral cavity because they could incorporate drugs, proteins, and peptides with a polymeric vehicle that can form multicomponent meshes at a time. These fibers can be individual or encapsulated. They are also used as a reservoir when adding different components [75].

There are different techniques for the manufacture of fibers. Some examples would be fibrillation, electrospinning, jet gas, and nanolithography. It has been possible to successfully encapsulate different drugs, with the mixture between them and a polymer solution through the electrospinning process [76]. At the same time, it depends not only on the technique but also on the polymeric material used and the type of drugs to be incorporated [77]. The electrospinning technique would be defined as the formation of fibers by electrostatic processes, which uses electrical forces to produce polymeric fibers with ranges from 2 nm to some micrometers, using natural or synthetic polymer solutions [78]. This process offers a unique capacity for the manufacture of fibers on nanoscale and additional relatively controlled pore structure [79]. Another additional advantage is that smaller pores can be obtained and a surface area larger than regular fibers [80].

Different polymeric materials, both natural and synthetic, have been used for fiber construction, collagen, alginate, chitosan, polyurethane, polypropylene, and cellulose acetate, for the manufacture of matrices used as a vehicle for the controlled release of the drugs. These systems are designed to keep medications in the mouth and regulate their release at the site. The fabrication of fibers offers the possibility to recharge various drugs at once, the easy adaptation in the required sites, have biomechanical properties that can be adapted to the conditions, can be more specific, and have better adsorption. Also, a low cost and reproducible manufacturing process [81] make them a simple and promising option for the study of immunomodulatory drug release, and new developments are studying the immune response to fibers [82].

These matrices of fibers require interconnected pores which serve as the structural support of the cells, so that they can adhere, grow, proliferate, and exchange molecules. This also facilitates the passage of oxygen, nutrients, and waste products. Different polymeric materials and their copolymers have been used, which have been combined with bioceramics such as hydroxyapatite and tricalcium phosphate, to improve the mechanical properties, degradation, and bioactivity [83]. With electrospinning, it is possible to produce this type of fiber on a nanoscale and with a mesh of highly interconnected pores [84].

The combination of fibers obtained from polycaprolactone (PCL), as a biodegradable polymer, with hydroxyapatite (HPA) as a bioactive ceramic, offers versatile biological and mechanical properties. Additionally, PCL is approved by the FDA as a biodegradable material, nontoxic, and its degradation products, presenting itself as a slow degradation material [85]. This polymer is a good candidate for long-term implantable systems, in hard and soft tissues. Hydroxyapatite gives it hydrophilicity, in addition to improving its mechanical properties. The most important properties of this are excellent conductivity and good hydrophilicity, necessary in the oral environment [86]. In these scaffolds, human fibroblasts demonstrate adhesion, proliferation, and uniform distribution. In addition, it turned out to be nontoxic to cells, promoting their growth [87]. This would be one of the most important antecedents to think about using it for the treatment of periodontal disease, adding anti-inflammatory drugs and immunomodulators. In addition, it would be an implantable option in the oral cavity of simple procurement and manufacturing.

According to the limitations reported for periodontal regeneration as a need to overcome the sequelae of periodontal disease, it is proposed to rethink the use of scaffolds and biomaterials. It is important to develop biomaterial which could induce host regulatory profiles to achieve greater control of the disease, modulating local inflammation.

## 3. Conclusions

Damage caused by chronic inflammatory pathologies has been constantly investigated, and loaded immunomodulators in biomaterials could be a therapeutic alternative to be used to help control the disease process and its sequelae, as well as a therapeutic approach for the secondary effects of treatment. The range of vehicles and biomaterials used for this purpose is large, but faces several challenges, either due to biological or mechanical properties. They have become a subject of current research with a broad future.

Despite the promising uses of various biomaterials of natural and synthetic origin and the multiple studies of biocompatibility and beneficial effects in different physiological and healing processes, the evidence about the possible role in host immunomodulation is limited to clinical observations, which leaves an open window for a great field of research.

Each of the biomaterials used for the treatment of periodontal disease and the tissue damage it causes has advantages and disadvantages. Different modifications have been made to overcome this. There is a lot to investigate in this field because complete periodontal regeneration continues to be a challenge.

## Data Availability

The data used to support this study are included within the article and references.
